# Interpopulational and seasonal variation in the chemical signals of the lizard *Gallotia galloti*

**DOI:** 10.7717/peerj.3992

**Published:** 2017-12-05

**Authors:** Roberto García-Roa, Rodrigo Megía-Palma, Jesús Ortega, Manuel Jara, Pilar López, José Martín

**Affiliations:** 1 Department of Evolutionary Ecology, National Museum of Natural Sciences (MNCN-CSIC), Madrid, Spain; 2 Laboratory of Evolutionary Ecology of Adaptations, School of Life Sciences, University of Lincoln, Lincoln, United Kingdom

**Keywords:** Chemical signals, Island, Climate, Tocopherol, Steroids, Semiochemical, Scent

## Abstract

Communicative traits are strikingly diverse and may vary among populations of the same species. Within a population, these traits may also display seasonal variation. Chemical signals play a key role in the communication of many taxa. However, we still know far too little about chemical communication in some vertebrate groups. In lizards, only a few studies have examined interpopulational variation in the composition of chemical cues and signals and only one study has explored the seasonal effects. Here we sampled three subspecies of the Tenerife lizards (*Gallotia galloti*) and analyze the lipophilic fraction of their femoral gland secretions to characterize the potential interpopulational variation in the chemical signals. In addition, we assessed whether composition of these secretions differed between the reproductive and the non-reproductive season. We analyzed variations in both the overall chemical profile and the abundance of the two main compounds (cholesterol and vitamin E). Our results show interpopulational and seasonal differences in * G. gallotia* chemical profiles. These findings are in accordance with the high interpopulational variability of compounds observed in lizard chemical signals and show that their composition is not only shaped by selective factors linked to reproductive season.

## Introduction

Communication is one of the main challenges to be met by animals (Bradbury & Vehrencamp, 2011). The outstanding diversity of communicative traits lies in subsequent evolutionary processes of diversification that often trigger a wide repertoire of signals with a vast-range of functions (Espmark, Amundsen & Rosenqvist, 2000; Maynard Smith & Harper, 2003). Coloration (e.g., Keyser & Hill, 2000), movements (e.g., Peters, 2008), sounds (e.g., Llusia et al., 2013), vibrations (e.g., Hebets, 2004), electric stimuli (e.g., Dunlap, 2002) and chemicals (e.g., Bacquet et al., 2015) are some examples of signals that animals employ to interact. Multiple pieces of evidence show that signaling traits can differ among populations of the same species (Barquero, Peters & Whiting, 2015; Giery & Layman, 2015; Barbosa, Rebar & Greenfield, 2016) or even change within a population throughout the year as a result of seasonal effects because of the variability of climate or reproductive dynamics, among other factors. For example, a study in the sagebrush cricket (*Cyphoderris strepitan*) showed that nightly calling duration differed among seasons as a result of the cost associated to the sound emission during the reproductive season (Sakaluk & Snedden, 1990). Likewise, seasonality can alter visual signals, as was described in blue tits (*Parus caeruleus*), in which UV and blue ornaments differed between reproductive and non-reproductive periods (Örnborg et al., 2002).

Although Darwin Jr (1859) noted the importance of chemical communication in social and sexual interactions, the underlying factors of the staggering diversity of chemical signals used by organisms in social and sexual interactions are relatively unclear and have attracted considerable interest of the scientific community in the last decades (Mason, 1992; Johnston & Del Barco-Trillo, 2009; Wyatt, 2014; Apps, Weldon & Kramer, 2015; Wyatt, 2017). Environmental variables (e.g., Weber et al., 2016; Baeckens et al., 2017), trophic resources (e.g., Henneken et al., 2017) or physiological changes and hormonal levels (e.g., Kent et al., 2008) are examples of biotic and abiotic factors that may modulate the production and expression of these signals (Symonds & Elgar, 2008; Steiger, Schmitt & Schaefer, 2010). In this context, and given that many of the above-mentioned factors operate differently throughout the year, chemical signaling might also change under the effect of seasonality. However, the few studies that have addressed this question are primarily focused on insects (e.g., McNeil, 1991) or mammals (Johnston & Del Barco-Trillo, 2009), whereas lizards have been almost neglected (but see Alberts et al., 1992).

Lizards release chemical signals through feces, skin and specialized follicular glands (Mason, 1992; Weldon, Flachsbarth & Schulz, 2008; Mayerl, Baeckens & Van Damme, 2015; García-Roa et al., 2017a). Specifically, the lipophilic fraction of the chemical secretions produced by the femoral and precloacal follicular glands might play a key role in lizard recognition, hierarchy establishment, or mate choice (Cooper Jr & Steele, 1997; Martín & López, 2006; Carazo, Font & Desfilis, 2008; Font et al., 2012; Pruett et al., 2016). Their composition (i.e., the number and abundance of chemical compounds) can differ between species, and/or sexes (Khannoon et al., 2011; García-Roa et al., 2016a; García-Roa et al., 2016b; García-Roa et al., 2017b; Martín et al., 2017). Nevertheless, the interpopulational variation in these secretions remains only known for a few species (Runemark, Gabirot & Svensson, 2011; Gabirot, López & Martín, 2012; Martín et al., 2013; MacGregor et al., 2017) and examples of seasonal variation are scarce (but see Alberts et al., 1992). Indeed, the fact that many lizard species from temperate areas only produce secretions during the mating season (Martín & López, 2014; Martín & López, 2015) has promoted a huge bias in the characterization of their composition (Alberts, 1990; Escobar, Labra & Niemeyer, 2001; Khannoon et al., 2011; Khannoon, 2012; García-Roa et al., 2016b). Many regions around the world, however, have favorable climatic conditions for lizards during most of the year. In this scenario, it would not be surprising that lizards from these areas could produce chemical signals across different seasons.

In this work, we analyzed the femoral gland secretions of three subspecies of the insular Tenerife lizard (*Gallotia galloti*). This is a large lizard endemic to the Canary Islands (Spain) divided in four subspecies: *G.g. eisentrauti, G. g. galloti*, *G. g. palmae* and *G.g. insulanagae* (Richard & Thorpe, 2001). These lizards inhabit different islands of the Canary Achipelago: Tenerife Island (*G. g. eisentrauti* and *G. g. galloti*), La Palma Island (*G. g. palmae*), and the small islets of Roque de Anaga (*G. g. insulanagae*). In addition, while *G. g. eisentrauti* and *G. g. palmae* inhabit cloudy and wet densely vegetated forest areas in northern Tenerife and La Palma respectively, *G. g. galloti* lives in dry and sunny semidesert areas in the south and the center of Tenerife (Thorpe & Brown, 1989; Bohórquez-Alonso & Molina-Borja, 2014). Hence, the divergent ecological conditions of each area might modulate the expression of chemical signals, as it has been described for visual ornaments (Thorpe & Brown, 1989). We investigated potential differences in the composition of the femoral gland secretions among particular subspecies in the entire chemical profile and in the abundance of the two main compounds: cholesterol and vitamin E (=α-tocopherol). It has been hypothesized that these two compounds might have protective properties under different environmental conditions; cholesterol might protect semiochemicals in dry conditions and vitamin E in wet environments (Escobar et al., 2003; Weldon, Flachsbarth & Schulz, 2008; Martín & López, 2014). Since the environment varies considerably among subspecies (Thorpe & Brown, 1989; Bohórquez-Alonso & Molina-Borja, 2014), this could translate into different abundances of both compounds. Moreover, as this lizard species is active during the whole year, we also examined whether the femoral gland secretions differed between the reproductive and the non-reproductive seasons.

## Material and Methods

### Study sites

**Figure 1:**
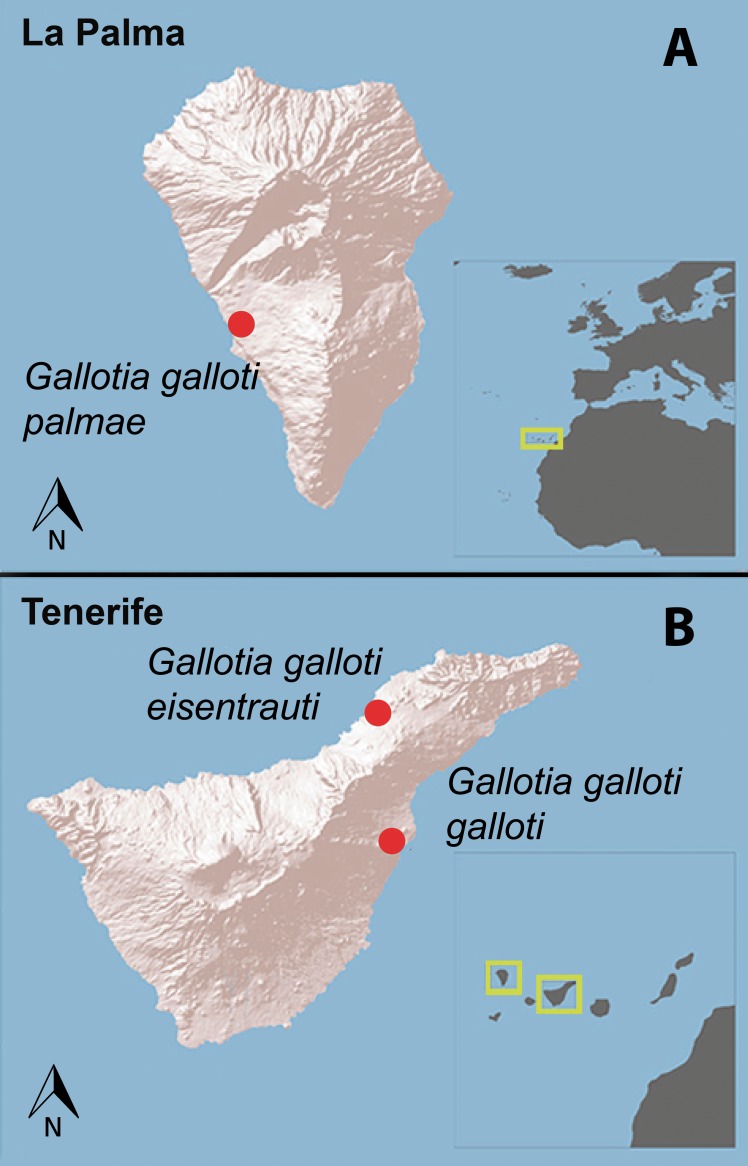
The islands of La Palma and Tenerife (Canary Islands, Spain). Red dots represent the sampling localities of *Gallotia galloti palmae*, *G. g. galloti* and *G. g. eisentrauti* male lizards in (A) La Palma and (B) Tenerife.

We conducted our study in two islands (Tenerife and La Palma) of the Canary Archipelago, Spain (Fig. 1). Because *G. galloti* lizards are active during the whole year, we focused on two different periods, spring (reproductive season; R) and winter (non-reproductive season; NR) (Salvador, 2015). In Tenerife, we visited two populations of *G. galloti*, in March (R) and December (NR) 2013. The *G. g. eisentrauti* population was located at El Pris in the North of the island (28°30′46″N, 16°25′4″W) and the *G. g. galloti* population was in Malpaís de Güímar, in the Southeast of the island (28°18′3″N, 16°23′49″W). The two sampled populations do not overlap in their distribution. Further, we studied a population of *G. g. palmae* close to El Pedregal at La Palma Island (28°37′13″N, 17°54′24″W) in April 2014 (R). Due to logistic reasons we could not sample this population in the non-reproductive season.

### Sampling and analysis of the femoral gland secretions

Field-work was carried out between 9:00 a.m. and 2:00 p.m. We captured a total of 30 males, *G. g. eisentrauti* (7), *G. g. galloti* (7) and *G. g. palmae* (7) in April, and *G. g. eisentrauti* (5) and *G. g. galloti* (4) in December, by using pitfall traps baited with banana and tomato (Oppliger, Vernet & Baez, 1999). All of the studied lizards were adults (snout-to-vent length range: *G. g. eisentrauti*, 92–130 mm; *G. g. galloti*, 92–125 mm; *G. g. palmae,* 100–120 mm). Traps were hidden next to bushes and rocks to avoid exposure to direct sunlight. We checked traps every 15 min to avoid overheating of captured lizards. Due to the high air temperatures in the islands, lizards were transported within individual cotton bags to a cool room. Ethics clearance and permits for this research were obtained from Cabildo Insular de La Palma (sampling permit: 201302/030-A/OT-098/2013) and Tenerife (13796/JBFT/CPA/AFF 97/13, 2013-00484). We collected femoral glands secretions of males to analyze and describe their chemical composition. Secretions were extracted by gently pressing glands and were introduced in glass vials with glass inserts and teflon-lined stoppers. In order to obtain blank controls, we used the same procedure without collecting secretion. Samples were stored at −20 °C until analyses. Lizards were healthy and we released them within the next 24 h in the same place where they had been captured.

We analyzed secretion using a Finnigan-ThermoQuest Trace 2000 gas chromatograph (GC), fitted with a column (5% diphenyl/95% dimethylsiloxane; Supelco, Equity-5, 30 m length × 0.25 mm ID, 0.25-µm film thickness), and a Finnigan-ThermoQuest Trace mass spectrometer (MS) as a detector. We operated in splitless analysis mode. We analyzed 2 µl of each sample dissolved in 200 µl of n-hexane (99%; obtained from JT Baker, Deventer, The Netherlands). We used helium as the carrier gas during the injection (270 °C) and detection (250 °C) phases with a constant septum purge. The temperature analysis program began at 50 °C isothermal for 10 min, increased to 280 °C at a rate of 5 °C/min, and then isothermal (280 °C) for 20 min. Data recording began 7 min after the separation initiated using the Software XcaliburTM 1.4 (Thermo Fischer Scientific Inc., San Jose, CA, USA). Previous to the analytical process, we examined the repeatability of the GC-MS process with five consecutive injections of a standard compound (heptadecane) made in different days, being the relative standard deviation (RSD) always below 1 for retention times and 10 for peak areas, which ensures the repeatability of the analytical method. Because of the small amount of secretions that we could extract from each individual, samples were analyzed only once, as it is usually done in similar studies (Alberts et al., 1992; Escobar, Labra & Niemeyer, 2001; Khannoon, 2012; Sáiz et al., 2017). Our analyses were focused on the lipophilic fraction of the femoral gland secretions and not in their proteins (Mangiacotti et al., 2016). Impurities identified in the control vial samples were not considered.

For the initial identification of compounds embodied in femoral gland secretions, we used the NIST/EPA/NIH 2002 computerized mass spectral library, through chemicals mass spectra comparison. When possible, identifications were confirmed by comparison of spectra and retention times with those of authentic standards (Sigma-Aldrich Chemical Co, St. Louis, MO, USA). Then, we calculated relative proportions of each compound determined as the percent of the total ion current (TIC) in each sample. For the comparison of overall chemical profiles (i.e., list of the identified compounds and their relative abundance per sample), we corrected the problem of non-independence of proportions using logit transformation of the proportion data by taking the natural logarithm of proportion / (1–proportion) (Aebischer, Robertson & Kenward, 1993).

### Statistical procedures

To assess potential differences in the chemical profile among subspecies, we used the software PRIMER v6.1.13 with the PERMANOVA+ v1.0.3 add-on package. We calculated the euclidean distances between every pair of individual samples to produce a resemblance matrix and then used a single factor permutational multivariate analysis of variance test (PERMANOVA) (McArdle & Anderson, 2001) based on the Euclidean resemblance matrix using 999 permutations. All identified compounds were included in the analysis. Possible differences were also analyzed with a canonical analysis of principal coordinates (CAP) (Anderson & Willis, 2003).

In addition, we tested for statistical differences between the reproductive and the non-reproductive season in *G. g. eisentrauti* and *G. g. galloti* populations. For this, we used a two-way PERMANOVA test and GLM analyses taking into account the effects of population, season and their interaction (Anderson & Willis, 2003). We confirmed both normality of data (Shapiro-Wilk’s test) and homogeneity of variances (Levene’s test) in all cases. Pairwise comparisons were performed with Tukey’s tests. All the statistical analyses were performed with R 3.2.2, SPSS 20.0.0 and STATISTICA v8.0 Software.

## Results

### Interpopulational differences in femoral gland secretions

The lipophilic fraction of the femoral gland secretions of *G. gallotia* lizards clearly differed among subspecies in the number, abundance and presence-absence of some compounds (Tables 1 and 2). Considering the three populations together, during the reproductive period, vitamin E (TIC = 34.91%) was the most abundant compound, closely followed by cholesterol (TIC = 24.32%).

**Table 1:** List of compounds identified in male femoral secretions of the lizard *Gallotia galloti.* Three subspecies were sampled: *G.g. eisentrauti, G.g. galloti and G.g. palmae* in the reproductive (R: april) and non-reproductive (NR: december) seasons. The relative proportion of each component was determined as the percent of the total ion current (TIC proportion) and reported as the mean (±1SD). Characteristic ions (m/z) are reported for unidentified compounds. RT: Retention time.

		*Gallotia galloti eisentrauti*						*Gallotia galloti galloti*						*Gallotia galloti palmae*		
		NR season (*n* = 5)			R season (*n* = 7)			NR season (*n* = 4)			R season (*n* = 7)			R season (*n* = 7)		
RT (min)	Compound	mean	±	SE	mean	±	SE	mean	±	SE	mean	±	SE	mean	±	SE
14.6	Nonanol	0.05	±	0.03	0.04	±	0.02	0.16	±	0.10	0.08	±	0.08	0.08	±	0.07
15.0	Decanal	0.01	±	0.05	0.01	±	0.01	0.02	±	0.02	0.02	±	0.03	–	–	–
17.4	2,4-Decadienal	0.01	±	0.01	0.02	±	0.02	–	–	–	–	–	–	0.01	–	0.02
19.6	Decanoic acid	0.01	±	0.01	0.02	±	0.02	–	–	–	–	–	–	–	–	–
20.0	Decanol	0.06	±	0.05	0.10	±	0.06	0.18	±	0.17	0.09	±	0.12	0.10	±	0.07
22.5	Undecanol	0.01	±	0.01	0.03	±	0.03	–	–	–	0.03	±	0.06	0.01	±	0.01
23.0	Dodecanal	0.01	±	0.01	0.01	±	0.01	–	–	–	–	–	–	–	–	–
24.3	Dodecanoic acid	–	–	–	0.07	±	0.09	–	–	–	–	–	–	–	–	–
24.8	Dodecanol	0.05	±	0.02	0.04	±	0.03	0.13	±	0.13	0.08	±	0.10	0.07	±	0.03
25.3	Tetradecanal	0.20	±	0.09	0.10	±	0.06	0.20	±	0.06	0.12	±	0.07	0.19	±	0.07
27.0	Tetradecanol	0.34	±	0.18	0.29	±	0.04	0.25	±	0.15	0.13	±	0.11	0.29	±	0.22
27.9	9-Hexadecenal	–	–	–	–	–	–	–	–	–	–	–	–	0.35	±	0.11
27.6	Pentadecanal	0.27	±	0.14	0.05	±	0.03	0.11	±	0.04	0.06	±	0.04	0.47	±	0.33
28.7	Tetradecanoic acid	0.01	±	0.01	0.19	±	0.14	–	–	–	0.04	±	0.04	–	–	–
29.2	Pentadecanol	0.17	±	0.03	0.04	±	0.02	0.13	±	0.10	0.08	±	0.12	–	–	–
29.7	Hexadecanal	1.23	±	0.61	0.75	±	0.38	0.62	±	0.24	0.27	±	0.23	1.28	±	0.94
30.3	6,10,14-Trimethyl-2-pentadecanone	0.01	±	0.01	0.03	±	0.02	0.01	±	0.01	0.02	±	0.03	–	–	–
30.7	Pentadecanoic acid	0.03	±	0.02	0.08	±	0.06	0.03	±	0.04	0.02	±	0.02	–	–	–
32.0	Hexadecanoic acid, methyl ester	–	–	–	–	–	–	–	–	–	–	–	–	0.78	+	0.40
31.2	Hexadecanol	1.24	±	0.72	0.39	±	0.24	1.12	±	0.80	0.44	±	0.34	–	–	–
31.4	2-Heptadecanone	0.20	±	0.12	0.05	±	0.03	0.08	±	0.03	0.05	±	0.03	0.17	±	0.11
31.8	Heptadecanal	0.23	±	0.08	0.03	±	0.03	0.11	±	0.07	0.08	±	0.15	–	–	–
32.3	9-Hexadecenoic acid	–	–	–	0.23	±	0.20	0.02	±	0.03	0.11	±	0.10	–	–	–
32.8	Hexadecanoic acid	0.44	±	0.78	4.06	±	1.20	0.79	±	1.46	2.96	±	2.04	0.89	±	0.76
33.2	Hexadecanoic acid, ethyl ester	0.08	±	0.07	0.11	±	0.11	0.04	±	0.04	0.77	±	1.58	0.09	±	0.07
33.5	Heptadecanol	0.01	±	0.01	0.07	±	0.07	–	–	–	0.02	±	0.03	–	–	–
33.7	Octadecanal	4.41	±	3.07	0.38	±	0.32	1.67	±	1.82	0.65	±	0.65	3.00	±	1.67
33.8	Hexadecanoic acid, 1-methylethyl ester	–	–	–	0.31	±	0.31	0.07	±	0.12	0.11	±	0.13	–	–	–
34.6	Heptadecanoic acid	–	–	–	0.08	±	0.03	–	–	–	0.06	±	0.06	–	–	–
35.0	Octadecanol	0.14	±	0.10	0.35	±	0.27	0.09	±	0.07	0.46	±	0.43	0.52	±	0.61
35.3	2-Nonadecanone	1.02	±	0.65	0.12	±	0.07	0.42	±	0.24	0.16	±	0.09	0.39	±	0.29
35.6	Nonadecanal	0.44	±	0.39	0.02	±	0.02	0.35	±	0.25	0.08	±	0.14	0.53	±	0.27
35.9	9,12-Octadecadienoic acid	0.01	±	0.01	1.49	±	1.11	–	–	–	1.62	±	1.38	0.02	±	0.04
36.0	9-Octadecenoic acid	0.10	±	0.18	2.33	±	1.08	–	–	–	4.48	±	7.91	0.19	±	0.50
36.3	9,12-Octadecadienoic acid, ethyl ester	–	–	–	0.27	±	0.50	–	–	–	0.37	±	0.50	–	–	–
36.4	Octadecanoic acid	0.29	±	0.52	2.00	±	1.10	0.03	±	0.05	2.29	±	1.35	0.61	±	1.12
36.8	Octadecanoic acid, ethyl ester	–	–	–	0.14	±	0.17	0.16	±	0.31	0.15	±	0.23	–	–	–
37.4	Eicosanal	0.18	±	0.14	0.30	±	0.16	0.16	±	0.13	0.10	±	0.12	0.27	±	0.23
38.7	Eicosanol	–	–	–	–	–	–	0.14	±	0.16	0.34	±	0.27	–	–	–
39.1	5,8,11,14-Eicosatetraenoic acid,ethyl ester	–	–	–	–	–	–	0.19	±	0.13	0.26	±	0.33	–	–	–
39.7	Eicosanoic acid	–	–	–	–	–	–	–	–	–	1.45	±	1.24	0.19	±	0.22
40.3	Eicosanoic acid, ethyl ester	–	–	–	–	–	–	–	–	–	0.65	±	0.79	–	–	–
46.7	Squalene	0.62	±	0.22	3.99	±	2.92	0.41	±	0.40	1.54	±	0.97	0.51	±	0.23
46.8	Cholesta-2,4-diene	0.24	±	0.25	0.32	±	0.36	0.16	±	0.23	0.02	±	0.03	0.06	±	0.07
47.0	Unidentified terpenoid	0.04	±	0.05	0.08	±	0.10	–	–	–	–	–	–	–	–	–
47.4	Cholesta-4,6-dien-3-ol	0.13	±	0.02	0.05	±	0.05	0.23	±	0.09	0.14	±	0.13	0.23	±	0.38
47.6	Cholesta-3,5-diene	0.34	±	0.16	0.49	±	0.34	0.33	±	0.20	0.31	±	0.29	0.32	±	0.27
49.5	γ-Tocopherol	–	–	–	0.01	±	0.01	–	–	–	0.01	±	0.01	0.03	±	0.08
50.2	Cholestanol	0.48	±	0.34	0.58	±	0.26	0.69	±	0.55	0.70	±	0.65	4.45	±	11.21
50.7	Cholesterol	51.92	±	14.07	19.35	±	6.65	54.21	±	6.25	41.98	±	13.32	11.65	±	4.39
50.8	α-Tocopherol (Vitamin E)	18.76	±	9.32	41.71	±	11.77	24.59	±	6.79	19.97	±	9.66	43.07	±	14.23
51.3	Cholestan-3-one	0.16	±	0.15	0.24	±	0.16	–	–	–	–	–	–	–	–	–
51.4	Ergosta-5,22-dien-3-ol	0.33	±	0.34	0.33	±	0.25	1.16	±	0.93	0.74	±	0.73	0.54	±	0.55
51.9	Ergosterol	0.16	±	0.12	0.19	±	0.26	–	–	–	–	–	–	1.10	±	1.93
52.1	Campesterol	7.71	±	2.21	4.58	±	2.83	3.52	±	1.63	4.84	±	2.40	12.38	±	17.31
52.3	Cholest-4-en-3-one	1.53	±	0.64	1.55	±	0.77	1.56	±	0.87	1.18	±	0.95	2.25	±	2.09
52.5	Stigmasterol	0.26	±	0.17	0.33	±	0.29	0.14	±	0.17	0.29	±	0.21	0.95	±	1.28
52.7	Cholesta-4,6-dien-3-one	0.33	±	0.06	0.30	±	0.21	0.57	±	0.28	0.29	±	0.22	0.36	±	0.30
53.0	Stigmasterol derivative?	0.05	±	0.10	0.13	±	0.22	–	–	–	–	–	–	0.82	±	1.59
53.2	Sitosterol	3.68	±	1.61	4.40	±	1.28	2.87	±	1.96	3.36	±	1.95	4.99	±	3.19
53.3	Stigmastanol	0.93	±	0.50	0.50	±	0.49	0.54	±	1.08	0.38	±	0.37	0.34	±	0.84
53.8	Unid. steroid(143,157,211,253,353,380,412)	0.01	±	0.02	0.28	±	0.52	–	–	–	–	–	–	–	–	–
53.8	Cholest-5-en-3-one	–	–	–	–	–	–	–	–	–	–	–	–	0.09	±	0.17
53.9	Hexadecanoic acid, ethenyl ester	0.13	±	0.08	1.13	±	0.30	0.16	±	0.10	0.26	±	0.24	0.15	±	0.24
54.1	Hexadecyl 9-hexadecenoate	0.06	±	0.07	0.28	±	0.22	–	–	–	–	–	–	–	–	–
55.3	Cholest-4-ene-3,6-dione	–	–	–	–	–	–	–	–	–	–	–	–	0.79	±	0.87
55.9	Octadecyl 9-hexadecenoate	–	–	–	0.44	±	0.37	0.02	±	0.04	0.16	±	0.15	–	–	–
56.2	Octadecyl hexadecanoate	0.04	±	0.57	1.12	±	1.52	0.48	±	0.48	2.05	±	3.48	–	–	–
56.4	9-Octadecenyl hexadecanoate	–	–	–	–	–	–	–	–	–	–	–	–	0.12	±	0.20
56.9	Octadecanoic acid, ethenyl ester	–	–	–	–	–	–	0.23	±	0.27	0.48	±	0.40	0.55	±	0.54
57.6	Eicosyl hexadecenoate	–	–	–	–	–	–	0.19	±	0.19	0.37	±	0.43	1.26	±	1.14
59.1	Unidentified waxy ester?	–	–	–	–	–	–	–	–	–	–	–	–	0.27	±	0.32
59.4	9-Octadecenyl 9-hexadecenoate	0.04	±	0.08	0.31	±	0.35	–	–	–	–	–	–	0.26	±	0.17
60.5	9-Octadecenyl 9-octadecenoate	–	–	–	–	–	–	0.24	±	0.28	1.16	±	1.40	–	–	–
60.7	9-Octadecenyl octadecanoate	–	–	–	–	–	–	–	–	–	–	–	–	0.62	±	1.02
60.9	Octadecyl octadecanoate	0.01	±	0.02	0.75	±	1.07	–	–	–	–	–	–	0.57	±	0.61
61.2	Unidentified waxy ester?	0.35	±	0.60	0.68	±	0.81	0.39	±	0.31	0.28	±	0.35	0.31	±	0.35
65.4	Unid. ester of 9-hexadecenoic acid	0.03	±	0.04	0.39	±	0.22	0.04	±	0.09	0.04	±	0.07	0.06	±	0.09
65.9	Octadecyl eicosanoate	0.03	±	0.05	0.81	±	0.56	–	–	–	0.79	±	0.91	0.36	±	0.51
67.2	Unidentified waxy ester?	0.36	±	0.14	0.07	±	0.10	–	–	–	–	–	–	–	–	–

There were significant differences among the chemical profiles of the three subspecies during the reproductive season (PERMANOVA; pseudo *F*_2,18_ = 48.99, *P* = 0.001). The pairwise comparisons showed significant differences in all cases (permutation tests; 6.20 < *t* < 7.34, *P* = 0.002 for all). The CAP analysis classified 95.2% of individuals within the correct subspecies in accordance with their chemical profiles, using leave-one-out cross-validation and m = 2 axes (${\mathrm{\delta }}_{1}^{2}=0.98$, *P* < 0.001).

**Figure 2:**
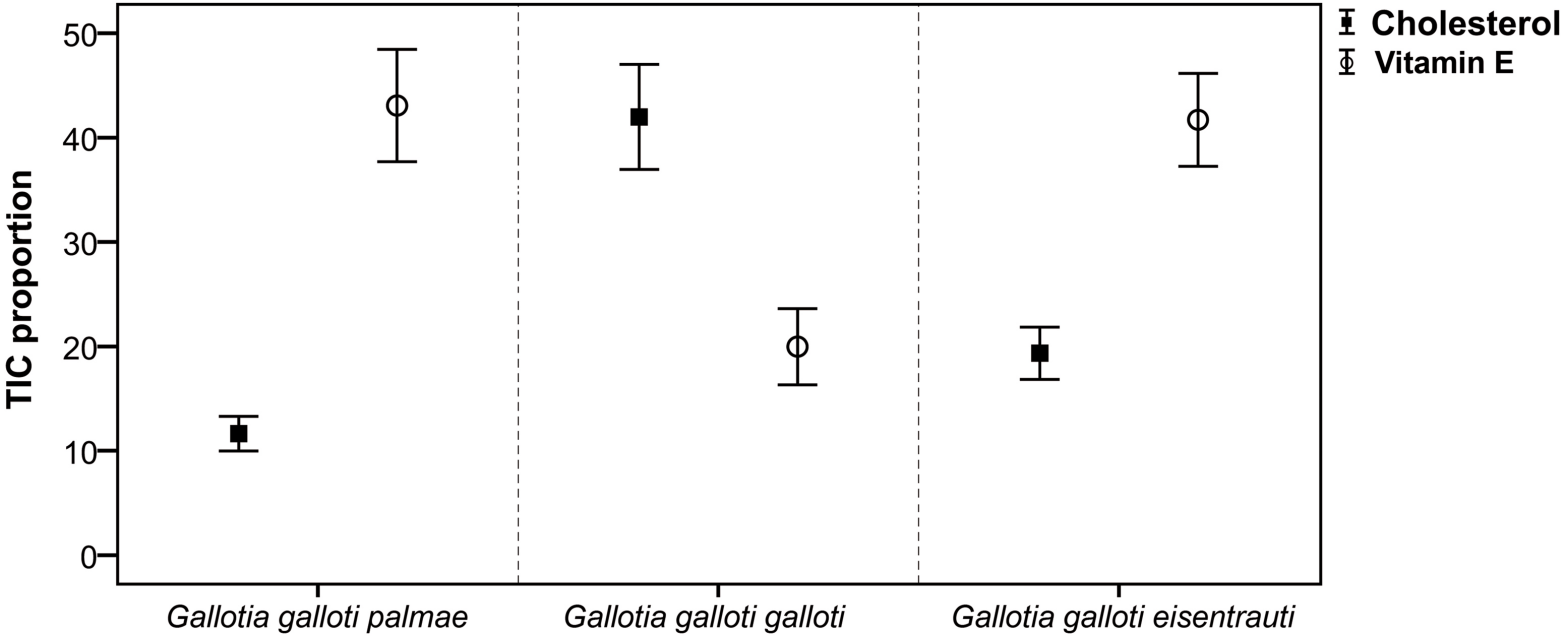
Comparison of the total ion current proportions (TIC) of cholesterol and vitamin E. TIC proportions (mean ± SE) from male femoral gland secretions of *G. g. palmae, G. g. galloti* and *G. g. eisentrauti* populations sampled in the reproductive season (April).

Focusing on cholesterol secreted in the reproductive season, we found significant differences among subspecies (GLM; *F*_2,18_ = 22.30, *P* < 0.001). However, the pairwise comparisons showed that *G. g. galloti* differed significantly from *G. g. eisentrauti* (Tukey’s tests, *P* < 0.01) and *G. g. palmae* (*P* < 0.001), but the difference between *G. g. eisentrauti* and *G. g. palmae* was not significant (*P* = 0.07) (Fig. 2). We observed a similar pattern in vitamin E, which differed significantly among subspecies (GLM; *F*_2,18_ = 8.51, *P* < 0.01). However, while *G. g. galloti* showed significant differences in the abundance of vitamin E with *G. g. eisentrauti* (Tukey’s tests, *P* < 0.01) and *G. g. palmae* (*P* < 0.01), *G. g. eisentrauti* and *G. g. palmae* did not significantly differ (*P* = 0.98).

**Table 2:** Percentage of semiochemicals found in male femoral secretions of *Gallotia galloti* lizards. Three subspecies were sampled: *G.g. eisentrauti, G.g. galloti and G.g. palmae* male lizards in the reproductive (R: april) and non-reproductive (NR: december) seasons.

	*Gallotia galloti eisentrauti*		*Gallotia galloti galloti*		*Gallotia galloti palmae*
	NR season (*n* = 5)	R season (*n* = 7)	NR season (*n* = 4)	R season (*n* = 7)	R season (*n* = 7)
Number of compounds	58	66	49	60	53
Alcohols	2.07	1.35	2.19	1.74	1.09
Aldehydes	6.99	1.67	3.24	1.37	6.11
Carboxylic acids and their esters	0.97	11.38	1.32	15.32	2.77
Ketones	1.23	0.20	0.5	0.22	0.57
Squalene and other terpenoids	0.66	4.07	0.41	1.54	0.50
Steroids	68.28	33.63	65.98	54.23	41.33
Waxy esters	1.04	5.98	1.76	5.60	4.53
Tocopherols	18.76	41.72	24.6	19.98	43.1

### Seasonal differences in femoral gland secretions

**Figure 3:**
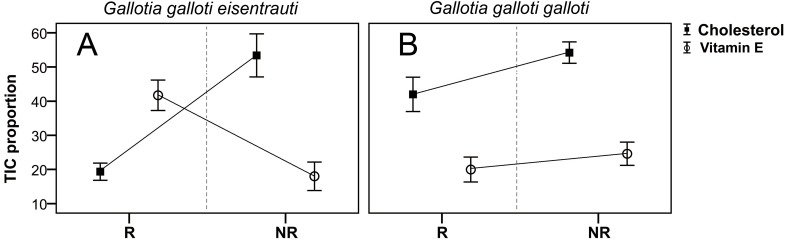
Seasonal differences in the total ion current proportions (TIC) of cholesterol and vitamin E. TIC proportions (mean ± SE) from male femoral gland secretions of *G. g. galloti* and *G. g. eisentrauti* populations sampled in the (A) reproductive (R: April) and (B) non-reproductive (NR: December) seasons.

We found seasonal variation in the composition of the femoral gland secretions between *G. g. eisentrauti* and *G. g. galloti* populations (two-way PERMANOVA; population: pseudo *F*_1,19_ = 73.38, *P* < 0.001; season: pseudo *F*_1,19_ = 9.73, *P* < 0.01; interaction population × season: pseudo *F*_1,19_ = 1.99, *P* = 0.13). The CAP analyses classified 100% of individuals within the correct subspecies (${\mathrm{\delta }}_{1}^{2}=0.99$, *P* < 0.001) or season (${\mathrm{\delta }}_{1}^{2}=0.90$, *P* < 0.001) according to their chemical profiles. In addition, we found significant differences in the abundance of cholesterol between these two subspecies (GLM; *F*_1,19_ = 7.60, *P* = 0.01) and also between the reproductive and the non-reproductive seasons (GLM; *F*_1,19_ = 18.01, *P* < 0.0001); the interaction was significant (population × season: *F*_1,19_ = 6.69, *P* = 0.01) (Fig. 3). Pairwise comparisons showed that there were seasonal differences in the abundance of cholesterol in *G. g. eisentrauti* (Tukey’s tests, *P* < 0.001), but not in *G. g. galloti* (*P* = 0.37). Moreover, differences in cholesterol abundance between *G. g. eisentrauti* and *G. g. galloti* were only significant during the reproductive season (*P* < 0.01), but not in the non-reproductive season (*P* = 0.99). Regarding vitamin E, we did not find significant differences between subspecies or seasons (GLM; population: *F*_1,19_ = 1.95, *P* = 0.17; season: *F*_1,19_ = 3.80, *P* = 0.06); the interaction was significant (population × season: *F*_1,19_ = 11.15, *P* < 0.01). We observed significant seasonal differences in the amount of vitamin E in *G. g. eisentrauti* (Tukey’s tests, *P* < 0.01) but not in *G. g. galloti* (*P* = 0.77). Moreover, differences between both subspecies were significant during the reproductive season (*P* < 0.01), but not in the non-reproductive season (*P* = 0.60) (Fig. 3).

## Discussion

This study describes clear differences among three subspecies of *G. gallotia* lizards in the overall composition of their femoral gland secretions. Cholesterol and vitamin E were the two main compounds and their abundance also differed among subspecies. Interestingly, we also observed seasonal effects in the composition of secretions.

Few studies have examined and reported interpopulational differences in lizard chemical signals. For example, Escobar et al. (2003) found differences in precloacal secretions between two populations of *Liolaemus fabiani* lizards. These authors hypothesized that the variation could be ascribed to the different environmental conditions found in the two populations. Two recent works analyzing femoral gland secretions in insular lizards also showed interpopulational variability. In these studies, the hypothesis that climatic conditions might be shaping the composition of femoral gland secretions was also suggested (Runemark, Gabirot & Svensson, 2011; Martín et al., 2013). Climatic conditions could be an important factor modulating the composition of chemical signals, as it has been suggested not only for lizards (Martín & López, 2013; Heathcote et al., 2014; Martín et al., 2017; Baeckens et al., 2017), but also for other animals (Sentis et al., 2015; Boullis et al., 2016). The fact that *G. g. gallotia* inhabits dry habitats and secretes the highest level of cholesterol, whereas *G. g. eisentrauti* and *G. g. palmae* inhabit areas with higher humidity and their secretions bear the highest abundance of vitamin E, is in line with the hypothesis that cholesterol and vitamin E might protect the chemical secretions in dry and wet conditions, respectively (Escobar et al., 2003; Weldon, Flachsbarth & Schulz, 2008; Martín & López, 2014). However, other alternative selective factors influencing signal evolution have been posited, such as differences in predation pressure (e.g., Hughes, Kelley & Banks, 2012), trophic resources (e.g., Henneken et al., 2017), intrasexual selection (e.g., Ord, Blumstein & Evans, 2001), alternative “sexual strategies” (e.g., Pellitteri-Rosa et al., 2014), population density (e.g., Fowler-Finn, Cruz & Rodríguez, 2017), use of alternative signaling modalities (Johnstone, 1996), the degree of interspecific competition (Losos, 2009), or habitat use (e.g., Alberts, 1992). We cannot therefore ensure that, in our study, the chemical profile variation among populations is totally driven by climatic variables. The use of a single population for each climatic region hampers an accurate correlation between chemical profiles and climate.

An intriguing and neglected area in the field of lizard chemical communication is to find out whether chemical signaling changes throughout the year, mainly because most of examined species inhabit temperate regions where lizards only produce chemical signals during the mating season. Alberts et al. (1992) offered the first evidence in this respect, showing that the femoral secretions of *Iguana iguana* have more lipids during the reproductive season and thus chemical signals changed between seasons. Several years later,  Martins et al. (2006) showed that the amount of secretion produced by follicular glands decreased during the non-reproductive period in comparison with the reproductive one. In that study, however, the information regarding chemical composition was lacking. Our results show that the composition of femoral secretions of *G. g. eisentrauti* and *G. g. galloti* differed considerably among seasons. Nevertheless, the analyses of the two most abundant compounds (cholesterol and vitamin E) showed seasonal variation in *G. g. eisentrauti*, but not in *G. g. galloti*. The effect of seasonality in the production and expression of these compounds might be variable. For instance, some compounds must be acquired through the diet (Weldon, Flachsbarth & Schulz, 2008) and therefore, changes in dietary availability could produce a higher or lesser degree of their expression in femoral gland secretions (García-Roa et al., 2017c). Recent research hypothesize that several compounds may be also under a trade-off between being used in metabolism functions or in chemical signaling (Kopena et al., 2011; Kopena, López & Martín, 2014b; Kopena, López & Martín, 2014a; Martín & López, 2015). If dietary availability and metabolic requirements of these compounds differ between seasons, this might also affect to the secreted lipophilic fraction. This could happen with vitamin E which is used in metabolic processes. In addition, health state (e.g., parasites, immune condition) or the endocrine profile (e.g., testosterone or corticosterone levels) may be also determinant variables affecting chemical signals in different seasons (Martín & López, 2015).

In Summary, we showed quantitative and qualitative interpopulational and seasonal differences in the femoral gland secretions of the insular lizard *G. galloti*. Although we could not tease apart the specific factors underlying this variation, our study provides an encouraging scenario to explore whether climate plays a key role shaping the chemical signals of *G. gallotia* subspecies. Future research should replicate the effects of climatic conditions on a widespread species over different climatic regions. Finally, given that *Gallotia* lizards may use other sensory channels to transfer information (e.g., visual or acoustic), integrative analyses that consider other modes of communication would provide a global view of how these lizards communicate in a multimodal context.

##  Supplemental Information

10.7717/peerj.3992/supp-1 Data S1 Raw data

## Additional Information and Declarations

### Data Availability

The following information was supplied regarding data availability: The raw data has been uploaded as Data S1.

## References

[ref-1] Aebischer NJ, Robertson PA, Kenward RE (1993). Compositional analysis of habitat use from animal radio-tracking data. Ecology.

[ref-2] Alberts AC (1990). Chemical properties of femoral gland secretions in the desert iguana, *Dipsosaurus dorsalis*. Journal of Chemical Ecology.

[ref-3] Alberts AC (1992). Pheromonal self-recognition in Desert iguanas. Copeia.

[ref-4] Alberts AC, Sharp TR, Werner DI, Weldon PJ (1992). Seasonal variation of lipids in femoral gland secretions of male green iguanas (*Iguana iguana*). Journal of Chemical Ecology.

[ref-5] Anderson MJ, Willis TJ (2003). Canonical analysis of principal coordinates: a useful method of constrained ordination for ecology. Ecology.

[ref-6] Apps PJ, Weldon PJ, Kramer M (2015). Chemical signals in terrestrial vertebrates: search for design features. Natural Product Reports.

[ref-7] Bacquet P, Brattström O, Wang H-L, Allen C, Löfstedt C, Brakefield PM, Nieberding CM (2015). Selection on male sex pheromone composition contributes to butterfly reproductive isolation. Proceedings of the Royal Society B: Biological Sciences.

[ref-8] Baeckens S, Martín J, García-Roa R, Pafilis P, Huyghe K, Van Damme R (2017). Environmental conditions shape the chemical signal design of lizards. Functional Ecology.

[ref-9] Barbosa F, Rebar D, Greenfield M (2016). Female preference functions drive interpopulation divergence in male signalling: call diversity in the bushcricket *Ephippiger diurnus*. Journal of Evolutionary Biology.

[ref-10] Barquero MD, Peters R, Whiting MJ (2015). Geographic variation in aggressive signalling behaviour of the Jacky dragon. Behavioral Ecology and Sociobiology.

[ref-11] Bohórquez-Alonso ML, Molina-Borja M (2014). Reflectance of sexually dichromatic UV-blue patches varies during the breeding season and between two population of *Gallotia galloti* (Squamata: Lacertidae). Biological Journal of the Linnean Society.

[ref-12] Boullis A, Detrain C, Francis F, Verheggen FJ (2016). Will climate change affect insect pheromonal communication?. Current Opinion in Insect Science.

[ref-13] Bradbury JW, Vehrencamp SL (2011). Principles of animal communication.

[ref-14] Carazo P, Font E, Desfilis E (2008). Beyond ‘nasty neighbours’ and ‘dear enemies’? Individual recognition by scent marks in a lizard (*Podarcis hispanica*). Animal Behaviour.

[ref-15] Cooper Jr WE, Steele LJ (1997). Pheromonal discrimination of sex by male and female leopard geckos (*Eublepharis macularius*). Journal of Chemical Ecology.

[ref-16] Darwin Jr C (1859). On the origins of species by means of natural selection.

[ref-17] Dunlap KD (2002). Hormonal and body size correlates of electrocommunication behavior during dyadic interactions in a weakly electric fish, *Apteronotus leptorhynchus*. Hormones and Behavior.

[ref-18] Escobar CM, Escobar CA, Labra A, Niemeyer HM (2003). Chemical composition of precloacal secretions of two *Liolaemus fabiani* populations: are they different?. Journal of Chemical Ecology.

[ref-19] Escobar CA, Labra A, Niemeyer HM (2001). Chemical composition of precloacal secretions of *Liolaemus* lizards. Journal of Chemical Ecology.

[ref-20] Espmark Y, Amundsen T, Rosenqvist G (2000). Animal signals: signalling and signal design in animal communication.

[ref-21] Font E, Barbosa D, Sampedro C, Carazo P (2012). Social behavior, chemical communication, and adult neurogenesis: studies of scent mark function in *Podarcis* wall lizards. General and Comparative Endocrinology.

[ref-22] Fowler-Finn K, Cruz D, Rodríguez R (2017). Local population density and group composition influence the signal-preference relationship in *Enchenopa treehoppers* (Hemiptera: Membracidae). Journal of Evolutionary Biology.

[ref-23] Gabirot M, López P, Martín J (2012). Interpopulational variation in chemosensory responses to selected steroids from femoral secretions of male lizards, *Podarcis hispanica*, mirrors population differences in chemical signals. Chemoecology.

[ref-24] García-Roa R, Cabido C, López P, Martín J (2016b). Interspecific differences in chemical composition of femoral gland secretions between two closely related wall lizard species, *Podarcis bocagei* and *Podarcis carbonelli*. Biochemical Systematics and Ecology.

[ref-25] García-Roa R, Carreira S, López P, Martín J (2016a). Genders matters: sexual differences in chemical signals of *Liolaemus wiegmannii* lizards (Iguania, Liolaemidae). Biochemical Systematics and Ecology.

[ref-26] García-Roa R, Jara M, Baeckens S, López P, Van Damme R, Martín J, Pincheira-Donoso D (2017a). Macroevolutionary diversification of glands for chemical communication in squamate reptiles. Scientific Reports.

[ref-27] García-Roa R, Jara M, López P, Martín J, Pincheira-Donoso D (2017b). Heterogeneous tempo and mode of evolutionary diversification of compounds in lizard chemical signals. Ecology and Evolution.

[ref-28] García-Roa R, Sáiz J, Gómara B, López P, Martín J (2017c). Dietary constraints can preclude the expression of an honest chemical sexual signal. Scientific Reports.

[ref-29] Giery ST, Layman CA (2015). Interpopulation variation in a condition-dependent signal: predation regime affects signal intensity and reliability. The American Naturalist.

[ref-30] Heathcote RJ, Bell E, d’Ettorre P, While GM, Uller T (2014). The scent of sun worship: basking experience alters scent mark composition in male lizards. Behavioral Ecology and Sociobiology.

[ref-31] Hebets EA (2004). Attention-altering signal interactions in the multimodal courtship display of the wolf spider *Schizocosa uetzi*. Behavioral Ecology.

[ref-32] Henneken J, Goodger JQD, Jones TM, Elgar MA (2017). Diet-mediated pheromones and signature mixtures can enforce signal reliability. Frontiers in Ecology and Evolution.

[ref-33] Hughes NK, Kelley JL, Banks PB (2012). Dangerous liaisons: the predation risks of receiving social signals. Ecology Letters.

[ref-34] Johnston RE, Del Barco-Trillo J, Pfaff DW, Arnold AP, Etgen AM, Rubin RT, Fahrbach SE (2009). Communication by chemical signals: behavior, social recognition, hormones and the role of the vomeronasal and olfactory systems. Hormones, brain and behavior.

[ref-35] Johnstone RA (1996). Multiple displays in animal communication:backup signals’ andmultiple messages’. Philosophical Transactions of the Royal Society of London B: Biological Sciences.

[ref-36] Kent C, Azanchi R, Smith B, Formosa A, Levine JD (2008). Social context influences chemical communication in *D. melanogaster* males. Current Biology.

[ref-37] Keyser AJ, Hill GE (2000). Structurally based plumage coloration is an honest signal of quality in male blue grosbeaks. Behavioral Ecology.

[ref-38] Khannoon ER (2012). Secretions of pre-anal glands of house-dwelling geckos (Family: Gekkonidae) contain monoglycerides and 1, 3-alkanediol. A comparative chemical ecology study. Biochemical Systematics and Ecology.

[ref-39] Khannoon ER, Flachsbarth B, El-Gendy A, Mazik K, Hardege JD, Schulz S (2011). New compounds, sexual differences, and age-related variations in the femoral gland secretions of the lacertid lizard *Acanthodactylus boskianus*. Biochemical Systematics and Ecology.

[ref-40] Kopena R, López P, Martín J (2014a). What are carotenoids signaling? Immunostimulatory effects of dietary vitamin E, but not of carotenoids, in Iberian green lizards. Naturwissenschaften.

[ref-41] Kopena R, López P, Martín J (2014b). Relative contribution of dietary carotenoids and vitamin E to visual and chemical sexual signals of male Iberian green lizards: an experimental test. Behavioral Ecology and Sociobiology.

[ref-42] Kopena R, Martín J, López P, Herczeg G (2011). Vitamin E supplementation increases the attractiveness of males’ scent for female European green lizards. PLOS ONE.

[ref-43] Llusia D, Márquez R, Beltrán JF, Benítez M, Do Amaral JP (2013). Calling behaviour under climate change: geographical and seasonal variation of calling temperatures in ectotherms. Global Change Biology.

[ref-44] Losos JB (2009). Lizards in an evolutionary tree: ecology and adaptive radiation of anoles.

[ref-45] MacGregor HE, Lewandowsky RA, d’Ettorre P, Leroy C, Davies NW, While GM, Uller T (2017). Chemical communication, sexual selection, and introgression in wall lizards. Evolution.

[ref-46] Mangiacotti M, Fumagalli M, Scali S, Zuffi MAL, Cagnone M, Salvini R, Sacchi R (2016). Inter-and intra-population variability of the protein content of femoral gland secretions from a lacertid lizard. Current Zoology.

[ref-47] Martín J, López P (2006). Links between male quality, male chemical signals, and female mate choice in Iberian rock lizards. Functional Ecology.

[ref-48] Martín J, López P (2013). Effects of global warming on sensory ecology of rock lizards: increased temperatures alter the efficacy of sexual chemical signals. Functional Ecology.

[ref-49] Martín J, López P, Rheubert JL, Siegel DS, Trauth SE (2014). Pheromones and chemical communication in lizards. Reproductive biology and phylogeny of lizards and tuatara.

[ref-50] Martín J, López P (2015). Condition-dependent chemosignals in reproductive behavior of lizards. Hormones and Behaviour.

[ref-51] Martín J, López P, Garrido M, Pérez-Cembranos A, Pérez-Mellado V (2013). Inter-island variation in femoral secretions of the Balearic lizard, *Podarcis lilfordi* (Lacertidae). Biochemical Systematics and Ecology.

[ref-52] Martins EP, Ord TJ, Slaven J, Wright JL, Housworth EA (2006). Individual, sexual, seasonal, and temporal variation in the amount of sagebrush lizard scent marks. Journal of Chemical Ecology.

[ref-53] Martín J, Zamora-Camacho FJ, Reguera S, López P, Moreno-Rueda G (2017). Variations in chemical sexual signals of *Psammodromus algirus* lizards along an elevation gradient may reflect altitudinal variation in microclimatic conditions. The Science of Nature.

[ref-54] Mason RT (1992). Reptilian pheromones. Biology of the Reptilia.

[ref-55] Mayerl C, Baeckens S, Van Damme R (2015). Evolution and role of the follicular epidermal gland system in non-ophidian squamates. Amphibia-Reptilia.

[ref-56] Maynard Smith J, Harper D (2003). Animal signals: oxford series in ecology and evolution.

[ref-57] McArdle BH, Anderson MJ (2001). Fitting multivariate models to community data: a comment on distance-based redundancy analysis. Ecology.

[ref-58] McNeil JN (1991). Behavioral ecology of pheromone-mediated communication in moths and its importance in the use of pheromone traps. Annual Review of Entomology.

[ref-59] Oppliger A, Vernet R, Baez M (1999). Parasite local maladaptation in the Canarian lizard *Gallotia galloti* (Reptilia: Lacertidae) parasitized by haemogregarian blood parasite. Journal of Evolutionary Biology.

[ref-60] Ord TJ, Blumstein DT, Evans CS (2001). Intrasexual selection predicts the evolution of signal complexity in lizards. Proceedings of the Royal Society of London B: Biological Sciences.

[ref-61] Örnborg J, Andersson S, Griffith SC, Sheldon BC (2002). Seasonal changes in a ultraviolet structural colour signal in blue tits, *Parus caeruleus*. Biological Journal of the Linnean Society.

[ref-62] Pellitteri-Rosa D, Martín J, López P, Bellati A, Sacchi R, Fasola M, Galeotti P (2014). Chemical polymorphism in male femoral gland secretions matches polymorphic coloration in common wall lizards (*Podarcis muralis*). Chemoecology.

[ref-63] Peters RA (2008). Environmental motion delays the detection of movement-based signals. Biology Letters.

[ref-64] Pruett JA, Zúñiga-Vega JJ, Campos SM, Soini HA, Novotny MV, Vital-García C, Martins EP, Hews DK (2016). Evolutionary interactions between visual and chemical signals: chemosignals compensate for the loss of a visual signal in male *Sceloporus* lizards. Journal of Chemical Ecology.

[ref-65] Runemark A, Gabirot M, Svensson E (2011). Population divergence in chemical signals and the potential for premating isolation between islet-and mainland populations of the Skyros wall lizard (*Podarcis gaigeae*). Journal of Evolutionary Biology.

[ref-66] Sáiz J, García-Roa R, Martín J, Gómara B (2017). Fast, sensitive, and selective gas chromatography tandem mass spectrometric method for the target analysis of chemical secretions from femoral glands in lizards. Journal of Chromatography A.

[ref-67] Sakaluk SK, Snedden WA (1990). Nightly calling durations of male sagebrush crickets, *Cyphoderris strepitans*: size, mating and seasonal effects. Oikos.

[ref-68] Salvador A, Salvador A, Marco A (2015). Lagarto Tizón—*Gallotia galloti*. Enciclopedia virtual de los vertebrados españoles.

[ref-69] Sentis A, Ramon-Portugal F, Brodeur J, Hemptinne JL (2015). The smell of change: warming affects species interactions mediated by chemical information. Global Change Biology.

[ref-70] Steiger S, Schmitt T, Schaefer HM (2010). The origin and dynamic evolution of chemical information transfer. Proceedings of the Royal Society B: Biological Sciences.

[ref-71] Symonds MR, Elgar MA (2008). The evolution of pheromone diversity. Trends in Ecology & Evolution.

[ref-72] Thorpe R, Brown R (1989). Microgeographic variation in the colour pattern of the lizard *Gallotia galloti* within the island of Tenerife: distribution, pattern and hypothesis testing. Biological Journal of the Linnean Society.

[ref-73] Weber MG, Mitko L, Eltz T, Ramírez SR (2016). Macroevolution of perfume signalling in orchid bees. Ecology Letters.

[ref-74] Weldon PJ, Flachsbarth B, Schulz S (2008). Natural products from the integument of nonavian reptiles. Natural Product Reports.

[ref-75] Wyatt TD (2014). Pheromones and animal behavior: chemical signals and signatures.

[ref-76] Wyatt TD (2017). Pheromones. Current Biology.

